# Supplementary material for: Content-rich biological network constructed by mining PubMed abstracts
Source: BMC Bioinformatics. 2004 Oct 8;5:147. doi: 10.1186/1471-2105-5-147 (PMC528731; doi:10.1186/1471-2105-5-147)
Supplement: Additional File 2 — The original results of the above study (non-essential files are deleted to keep the file size under the limit set by BMC bioinformatics). [file 1471-2105-5-147-S2.bz2 › chilibotAdditionalFile2/dip10/200ID8652557E689/html/GTF2H1_ERCC5.html]

 


 **GTF2H1** and **ERCC5** 
  
Found 29 abstracts in PubMed, retrieved 10.  
 

 What does Google say? 
 PDF only 
| .edu only 

---

**Interactive relationship** (e.g. stimulation, inhibition, etc)

**Inhibitory relationship**- Furthermore, we reveal that TFIIH  [ **GTF2H1** ]  inhibits the structure specific endonuclease activities of both XPG  [ **ERCC5** ]  and ERCC1 XPF, responsible for the 3 and 5 incision in NER, respectively.  Ref: 11141066 Biochemistry, 2001
**Neutral relationship**- Strong functional interactions of TFIIH  [ **GTF2H1** ]  with XPC and XPG  [ **ERCC5** ]  in human DNA nucleotide excision repair, without a preassembled repairosome.  Ref: 11259578 Mol Cell Biol, 2001
- Novel functional interactions between nucleotide excision DNA repair proteins influencing the enzymatic activities of TFIIH  [ **GTF2H1** ] , XPG  [ **ERCC5** ] , and ERCC1 XPF.  Ref: 11141066 Biochemistry, 2001
- At physiological ionic strength, TFIIH  [ **GTF2H1** ]  associated with significant amounts of XPC HR23B and XPG  [ **ERCC5** ]  but not other repair factors.  Ref: 11259578 Mol Cell Biol, 2001

**Non-interactive relationship** (e.g. studied together, co-existance, homology, etc.)

- Here we show that with a highly defined system of purified repair factors, DDB can greatly stimulate the excision reaction reconstituted with XPA, RPA, XPC.HR23B, TFIIH  [ **GTF2H1** ] , XPF.ERCC1 and XPG  [ **ERCC5** ] , up to 17 fold for CPDs and approximately 2 fold for 6 4 photoproducts 6 4PPs, indicating that no additional factor is required for the stimulation by DDB.  Ref: 11705987 J Biol Chem, 2002
- XPA and RPA, once forming a complex at the damage site, would likely work with TFIIH  [ **GTF2H1** ] , XPG  [ **ERCC5** ] , and ERCC1 XPF for dual incision.  Ref: 12486030 J Biol Chem, 2003
- In mammalian cells, the core factors involved in the damage recognition and incision steps of DNA nucleotide excision repair are XPA, TFIIH  [ **GTF2H1** ]  complex, XPC HR23B, replication protein A RPA, XPG  [ **ERCC5** ] , and ERCC1 XPF.  Ref: 11259578 Mol Cell Biol, 2001
- Here, we provide evidence that CSB is found not only in the nucleoplasm but also in the nucleolus within a complex CSB IP 150 that contains RNA pol I, TFIIH  [ **GTF2H1** ] , and XPG  [ **ERCC5** ]  and promotes efficient rRNA synthesis.  Ref: 12419226 Mol Cell, 2002
- Transcription coupled repair of 8 oxoguanine requirement for XPG  [ **ERCC5** ] , TFIIH  [ **GTF2H1** ] , and CSB and implications for Cockayne syndrome.Analysis of transcription coupled repair of oxidative lesions here reveals strand specific removal of 8 oxo guanine and thymine glycol both in normal human cells and xeroderma pigmentosum cells defective in nucleotide excision repair.  Ref: 10786832 Cell, 2000
- Human nucleotide excision repair is initiated by six repair factors XPA, RPA, XPC HR23B, TFIIH  [ **GTF2H1** ] , XPF ERCC1, and XPG  [ **ERCC5** ]  which sequentially assemble at sites of DNA damage and effect excision of damage containing oligonucleotides.  Ref: 12138203 Mol Cell Biol, 2002
- Core NER enzymes XPA, XPF, XPG  [ **ERCC5** ] , and TFIIH  [ **GTF2H1** ]  are essential for recovery.  Ref: 12531012 DNA Repair Amst , 2002
- These results point toward additional roles for TFIIH  [ **GTF2H1** ]  and ATP during NER distinct from a requirement for DNA unwinding in the regulation of the endonuclease activities of XPG  [ **ERCC5** ]  and ERCC1 XPF.  Ref: 11141066 Biochemistry, 2001
